# Advanced Diagnostic System and Introduction of Newborn Screening of Adrenoleukodystrophy and Peroxisomal Disorders in Japan

**DOI:** 10.3390/ijns7030058

**Published:** 2021-08-25

**Authors:** Nobuyuki Shimozawa, Shigeo Takashima, Hiroki Kawai, Kazuo Kubota, Hideo Sasai, Kenji Orii, Megumi Ogawa, Hidenori Ohnishi

**Affiliations:** 1 Life Science Research Center, Division of Genomics Research, Gifu University, Gifu 501-1193, Japan; staka@gifu-u.ac.jp (S.T.); kawai0427@gmail.com (H.K.); 2 Department of Pediatrics, Gifu University Graduate School of Medicine, Gifu 501-1194, Japan; kubotak.gif@gmail.com (K.K.); sasai@gifu-u.ac.jp (H.S.); kenjior-gif@umin.ac.jp (K.O.); ohnishih@gifu-u.ac.jp (H.O.); 3 Clinical Genetics Center, Gifu University Hospital, Gifu 501-1194, Japan; 4 Gifu Research Center for Public Health, Gifu 500-8148, Japan; megumi@koeiken.or.jp

**Keywords:** peroxisomal disorders, adrenoleukodystrophy, newborn screening, very-long-chain fatty acids, plasmalogen, phytanic acid, presymptomatic diagnosis, *ABCD1*, whole-exome sequencing, dried blood spot

## Abstract

We established a diagnostic system for adrenoleukodystrophy (ALD) and peroxisomal disorders (PD) over 35 years ago in Japan, and have diagnosed 237 families with ALD and more than 100 cases of PD other than ALD using biochemical and molecular analyses. In particular, since the only treatment for the cerebral form of ALD is hematopoietic stem cell transplantation at an early stage of onset, we have developed a protocol for the rapid diagnosis of ALD that can provide the measurements of the levels of very-long-chain fatty acids in the serum and genetic analysis within a few days. In addition, to improve the prognosis of patients with ALD, we are working on the detection of pre-symptomatic patients by familial analysis from the proband, and the introduction of newborn screening. In this review, we introduce the diagnostic and newborn screening approaches for ALD and PD in Japan.

## 1. Introduction

Peroxisomal disorders (PD) are inborn errors of metabolism that affect the metabolic function of peroxisomes (intracellular organelles), and can be divided into two groups: peroxisome biogenesis disorder (PBD) and single enzyme deficiency (SED) [1]. SED is caused by genetic abnormalities in proteins localized in the peroxisomes, and PBD is caused by genetic abnormalities in proteins involved in the transport of these proteins to the peroxisomes and in the biosynthesis of peroxisomal membranes. Zellweger syndrome (ZS) is a prototype of PD in which generalized peroxisomal functions, such as β-oxidation of fatty acids, α-oxidation of phytanic acid, and the biosynthesis of plasmalogen are impaired. Our discovery of the pathogenic gene for ZS in 1992 [2] has contributed to further elucidation of the molecular pathology and genetic diagnosis of PBD.

Adrenoleukodystrophy (ALD), the most common PD, is an X-linked inherited disease caused by a pathogenic mutation in the *ABCD1* gene and a dysfunction of its product, ABCD1, a peroxisomal membrane protein involved in the import of saturated very-long-chain fatty acids (VLCFA) into the peroxisomes, leading to impaired β-oxidation of saturated VLCFA. This results in the accumulation of saturated VLCFA in the tissues and plasma. ALD has various phenotypes (Table 1) that do not correlate with the genotypes; therefore, even if diagnosed before the onset of symptoms, the timing of the onset and prognosis cannot be predicted. The prognosis of the cerebral type of ALD is generally very poor, and many patients become bedridden within a few years. Hematopoietic stem cell transplantation (HSCT) is currently the only curative approach that can prevent the progression of brain degeneration; however, HSCT is only effective for patients in the early stages of cerebral ALD [3]. Therefore, not only prompt diagnosis after the onset of the disease, but also presymptomatic diagnosis, including newborn screening, is essential to prevent the progression of cerebral ALD.

As the only center for the diagnosis of PD in Japan, we have diagnosed many patients with PD through the diagnostic screening of peroxisomal metabolites and with biochemical and genetic analyses for 35 years. Since rapid diagnosis is especially essential for ALD, we established a diagnostic system that provides results within a few days. Nevertheless, many patients are diagnosed too late to receive HSCT at the appropriate time and have a grave prognosis.

In this review, we present our efforts to diagnose ALD and PD and introduce newborn screening in Japan.

## 2. Diagnostic System for ALD and PD in Japan

**Figure 1:**
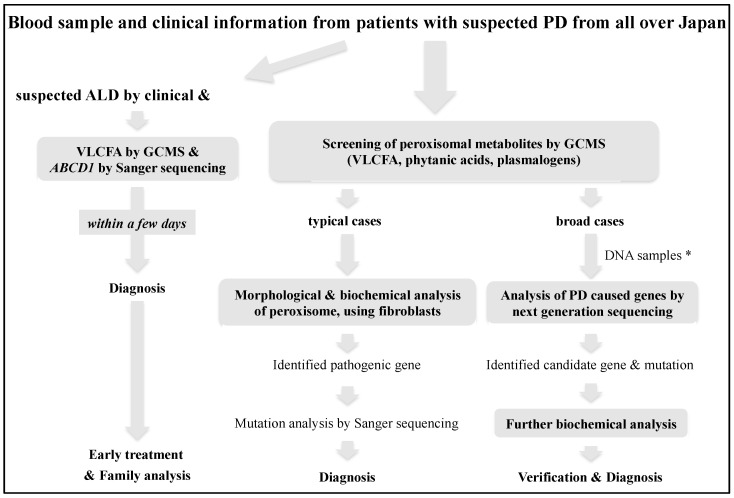
Diagnostic flowchart for adrenoleukodystrophy and peroxisomal disorders at Gifu University [8]. * collaborated with Hamamatsu University School of Medicine [7].

Initially, for diagnostic screening, we measured peroxisomal metabolites containing VLCFA, phytanic acids, and plasmalogen in the serum or plasma of patients by gas chromatography/mass spectrometry analysis (GCMS) [4], following which the disease-causing genes were detected by biochemical, morphological, and molecular analyses in typical cases [5]. However, in recent years, PD with broad phenotypes and newly identified genotypes have been reported due to improvements in the analytical techniques of peroxisomal metabolic function and the spread of whole-exome sequencing. Therefore, we now perform advanced diagnostic analysis for broad cases by more detailed lipid analysis, using liquid chromatography-mass spectrometry (LCMS) [6] and next-generation sequencing to interrogate relevant genes in the exome [7]. At present, it seems that the analysis of peroxisomal metabolites should be prioritized to confirm the diagnosis and obtain evidence that can predict the severity, pathogenesis, and treatment. However, comprehensive gene analysis may be prioritized when it becomes efficient in terms of time and cost in the foreseeable future, followed by biochemical analysis to verify the pathogenic significance. On the other hand, for the diagnosis of ALD, it is important to diagnose symptomatic cerebral ALD as soon as possible; therefore, we provide a confirmed diagnosis combining VLCFA and *ABCD1* mutation analyses within a few days (Figure 1) [8]. To confirm the pathogenic mutation, the ALD mutation database on the ALD information website (https://adrenoleukodystrophy.info/mutations-and-variants-in-abcd1 (accessed on 20 May 2021)) and our Japanese mutation data of 237 families are useful.

To date, we have diagnosed 83 Japanese patients with PBD, 5 with Acyl-CoA oxidase 1 deficiency, 13 with D-bifunctional protein deficiency, 2 with rhizomelic chondrodysplasia punctate (RCDP) type 2, 1 with RCDP type 3, and 237 Japanese families with ALD, including 99 patients with childhood cerebral ALD, 12 with adolescent cerebral ALD, 17 with adult cerebral ALD, 55 with adrenomyeloneuropathy, 6 with Olivo-Ponto-cerebellar ALD, 29 with Addison-only and 43 with pre-symptomatic ALD (Table 1), and 194 female carriers.

## 3. Efforts to Diagnose ALD before the Onset of Cerebral Type at Gifu University

**Figure 2:**
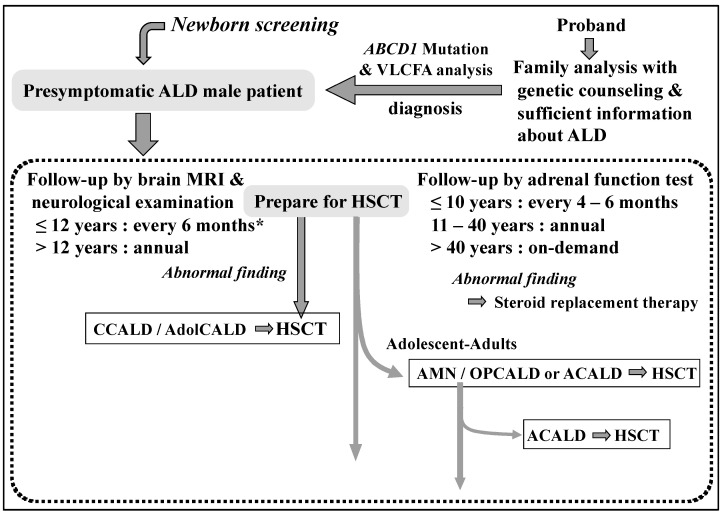
Follow-up flowchart for patients with pre-symptomatic adrenoleukodystrophy [8]. * start MRI follow-up at 12 to 18 months of age [13].

In cerebral ALD, the disease often progresses without patients receiving HSCT at the appropriate time because of the delay in diagnosis due to diverse and non-specific symptoms. Therefore, at the diagnosis of ALD patients, we encourage the family to understand the importance of pre-symptomatic diagnosis in ALD and recommend genetic counseling, after prioritizing the treatment of the patients. Furthermore, no patient with the Addison-only type of ALD was reported in a nationwide survey in Japan in the 1990s [9]. Therefore, we have been promoting the importance of VLCFA tests for the differential diagnosis in men with Addison’s disease. Through these efforts, we diagnosed 29 male patients as Addison-only at diagnosis and 43 pre-symptomatic male patients, which led to appropriate follow-up (Figure 2) [8] based on guidelines and references [10,11,12]. More recently, with regard to the timing of starting the MRI follow-up, in order to examine more efficiently and with less burden, it has been suggested that the first examination be done at 12 to 18 months of age, the second after 1 year, and then every 6 months between 3 and 12 years of age [13]. These follow-up efforts lead to steroid replacement therapy and HSCT at the appropriate time to improve the prognosis in male patients with ALD. In these circumstances, newborn screening for ALD has already begun in the USA and the Netherlands. Therefore, we have recently begun a pilot study for newborn screening of ALD in a limited region in order to accumulate a variety of evidence to gain logical rationale and social acceptance of disseminating it throughout Japan.

## 4. Introduction of the ALD Newborn System in the Gifu Prefecture

Nationwide mass screening of newborns in Japan is conducted for 20 diseases at public expense: congenital hypothyroidism, congenital adrenal hyperplasia, galactosemia, five disorders of amino acid metabolism (phenylketonuria, maple syrup urine disease, homocystinuria, citrullinemia type I, and argininosuccinic aciduria), seven disorders of organic acid metabolism (methylmalonic aciduria, propionic acidemia, isovaleric acidemia, methylcrotonylglycinuria, 3-hydroxy-3-methylglutaric aciduria, multiple carboxylase deficiency, and glutaric aciduria type 1), and five disorders of fatty acid β oxidation (medium-chain acyl-coenzyme A (CoA) dehydrogenase deficiency, very-long-chain acyl-CoA dehydrogenase deficiency, trifunctional protein deficiency, and carnitine palmitoyltransferase type I and II deficiency). Furthermore, newborn screening for several intractable diseases in which early diagnosis and treatment may be the optimal approach have already been performed in some groups in Japan, in addition to these 20 diseases. For example, a group at Kumamoto University has been undertaking newborn screening for lysosomal storage disorders, including Fabry disease, Pompe disease, Gaucher disease, and mucopolysaccharidosis type I and II since 2006 [14,15].

We initiated expanded newborn screening for ALD, in addition to Fabry disease, Pompe disease, mucopolysaccharidosis type I and II, primary immunodeficiencies (such as severe combined immunodeficiency and B-cell deficiency), and spinal muscular atrophy, in April 2021 as a pilot study with the approval of the Ethical Committee of Gifu University Graduate School of Medicine (2020-212), for children born in the Gifu Prefecture over a period of 4 years. This expanded screening is performed as a paid test for children of guardians who have been briefed and given informed consent at maternity hospitals or neonatal care centers. Dried blood spots (DBSs) are prepared at 4–6 days after birth, concurrently with the already-instituted newborn screening tests. Screening is conducted at the Gifu Research Center for Public Health, which has been providing the routine newborn screening.

For the screening of ALD, there are effective treatments for cerebral forms and adrenal insufficiency in male patients, and early diagnosis improves their prognosis as already described. On the other hand, for female carriers, the onset of disease occurs after reaching adulthood, and there are currently no methods to prevent or cure their symptoms. Family analysis of female carriers may improve prognosis by detecting pre-onset males. However, girls who are screened and diagnosed at birth as female carriers are not able to benefit from the prevention of health problems themselves. Therefore, in this study, we decided to record results only for babies who were determined to be boys based on the shape of their external genitalia at the hospital where the DBS was sampled.

Screening for ALD was performed by the measuring of C26:0 lysophosphatidylcholine (C26:0-LPC) and C24:0-LPC levels in DBSs from neonates by modifying the method described in the literature [16]. Using high-performance liquid chromatography-tandem mass spectrometry (HPLC-MS/MS) (LCMS-8500, Shimadzu, Kyoto, Japan), the step of flow injection analysis-tandem mass spectrometry (FIA-MS/MS) was omitted. A Waters XBridge BEH C18 XP column (2.1 mm × 50 mm, particle size 2.5 μm) preceded by a Waters BEH C18 XP VanGuard Pre-Column (2.1 mm × 5 mm, particle size 2.5 μm) was used for separation (Waters, Milford, MA, USA). The neonate screening kit (MS2 Screening OP, The Daiichi Kishimoto Clinical Laboratories, Sapporo, Japan and Siemens Healthineers, Erlangen, Germany) used for analysis took 2 min per sample. For boys who exceeded the cut-off value of C26:0-LPC or C24:0-LPC in the repeat test, we carefully explained the results to the guardians and requested to recollect the DBS sample, and then reanalyzed the sample by HPLC-MS/MS. The boys who exceeded the cut-off value in the repeat test with different DBS samples were referred to the Department of Pediatrics at Gifu University Hospital. The specialists in pediatric neurology and clinical genetics managed their precise diagnosis, and the results of VLCFA and *ABCD1* genetic analyses were provided within 5 days. When the male neonates were diagnosed with ALD, pediatric neurologists, pediatric endocrinologists, and pediatric blood transplant specialists worked together to provide appropriate follow-up and preparation for HSCT (Figure 2). Furthermore, we provided psychological counseling to the parents with the help of a clinical psychologist, in addition to genetic counseling in cooperation with clinical genetic counselors for at-risk patients in the family. When no *ABCD1* pathogenic variants were detected in positive patients, we proceeded to the diagnostic flowchart for PD at Gifu University (Figure 1).

Approximately 1000 boys were analyzed after the start of screening. No positive case has yet been detected: one false positive case was negative on retesting with a recollected DBS sample. This pilot study will include 28,000 boys expected to be born in the Gifu Prefecture over a 4-year period. While considering expansion to other prefectures during this period, we will accumulate various evidence and issues, such as the approximate incidence in the Gifu Prefecture, the accuracy and speed of both screening and definitive diagnoses, psychological counseling for parents, genetic counseling for family testing, and the validity of screening for boys alone to develop further discussion.

## 5. Discussion

We have been running the diagnostic center for ALD and PD in Japan for many years, and have diagnosed many cases in Japan. In particular, for patients with suspected cerebral ALD, we provide rapid diagnosis using VLCFA and genetic analyses within 3 working days. Furthermore, we recommend HSCT for patients at the appearance of abnormal magnetic resonance imaging (MRI) findings before the onset of cerebral symptoms to improve the prognosis of patients with cerebral ALD by expanding the analysis of the proband′s family and male patients with Addison′s disease.

In the USA, a screening method using DBS was developed in 2006 using C26:0-LPC [17]; newborn screening for both boys and girls that was initiated in the state of New York in December 2013 detected 45 patients with ALD (22 boys and 23 girls) among 700,000 newborns over 3 years. Newborn screening of ALD has spread across the USA after addition to the recommended uniform screening panel (RUSP) in February 2016 (https://adrenoleukodystrophy.info/clinical-diagnosis/ald-newborn-screening (accessed on 20 May 2021)). The reasons for including both sexes in the USA include the possibility that female carriers diagnosed by screening might lead to the detection of more pre-symptomatic male patients with ALD by expanding the analysis of the family. In addition, this can help in the early diagnosis of patients of both sexes with increased VLCFA who might have PD other than ALD [18]. In fact, some data have been reported from several states in the USA that include not only accurate birth rates of men and women, but also the detection of more ALD patients through family testing, and several patients with PD, by the screening of both boys and girls [19,20]. Furthermore, new evidence of increased C26:0-LPC levels in the neonatal period in patients with Aicardi–Goutieres syndrome (AGS) has been reported [21].

In Japan, newborn screening for ALD has been considered prudent because the prognosis cannot be predicted even if it is diagnosed in the neonatal period. The establishment of a newborn screening protocol for ALD has recently been discussed, given the achievements of newborn screening in the USA and the fact that approximately 40% of male patients with ALD develop cerebral type ALD by adulthood [9], in addition to the possibility of adrenal crisis due to latent adrenal insufficiency since childhood.

Under these circumstances, in the Netherlands, a stage-mediated system to screen only boys called “The X-Factor” has been developed, and newborn screening for ALD was commenced in October 2019 [22]. Only boys are screened for ALD in the Netherlands, in light of the Wilson and Jungner criteria [23]; the age of onset of myelopathy in female carriers is generally 40–60 years, and there is no effective treatment for the myelopathy; moreover, the onset of the cerebral type and adrenal insufficiency is extremely rare in female carriers of ALD.

The 20 diseases for which mass screenings are being conducted at public expense in Japan are autosomal recessive inherited diseases; there is little evidence of long-term evaluation of X-linked inherited diseases. Among them, newborn screening for Fabry disease, an X-linked inherited disease, has been performed for both boys and girls in some regions of Japan [14]. Female carriers of this disease can be as severely disabled as males, and enzyme replacement therapy has been developed, resulting in the possible improvement of prognosis by early diagnosis and treatment at the appropriate times. On the other hand, treatment for myopathy in female ALD carriers is limited to symptomatic therapy, and the cerebral form or adrenal insufficiency, for which appropriate treatment through early diagnosis can prevent health problems, hardly occurs in female carriers. Furthermore, epidemiological studies of female ALD carriers in Japan are insufficient; therefore, it has been thought that the risk of onset the disease in female carriers may be not so great.

In response to these circumstances, we have begun newborn screening in boys only that were born in the Gifu Prefecture as a pilot study with the approval of the Ethical Committee, after establishing a precise diagnostic protocol for screening positive boys and an appropriate follow-up protocol for diagnosed patients. The shape of the external genitalia judged by clinicians at the birth institution determines the sex of the neonate, not a gene test, as is the situation with the X-Factor program in the Netherlands [22]. Therefore, it is possible that male patients with disorders of sex development and those with Turner′s syndrome may be excluded in this study, which is an issue that should be considered in the future.

All infants that screen positive are referred to Gifu University, and ALD can be diagnosed within 5 days, and PD with increased VLCFA other than ALD, such as PBD, Acyl-CoA oxidase 1 deficiency, and D-bifunctional protein deficiency can be diagnosed promptly using our diagnostic system (Figure 1). In addition, AGS patients with increased C26:0-LPC levels are also expected to be provided with a prompt diagnosis. Information from the patient group has suggested that the most stressful time interval is between suspicion and confirmation of the diagnosis. Patients diagnosed with ALD are followed up appropriately by specialists in ALD, pediatric neurology, pediatric endocrinology, clinical genetics, blood transplantation, and neurology at Gifu University Hospital, to prevent the onset of cerebral type ALD and adrenal insufficiency and improve their prognosis (Figure 2). Furthermore, genetic counseling with the help of a clinical genetic counselor is provided not only for the parents, but also for at-risk patients in the family with their consent. While conducting pilot studies over the next four years, we plan to provide our precise diagnostic system to other prefectures when they begin screening for ALD. Furthermore, the expansion of this study to other prefectures is expected to lead to investigation of the natural history of male ALD patients from the neonatal period in Japan, as well as the elucidation of the pathogenesis of ALD using samples collected before and after the onset of the cerebral form of the patients with informed consent.

Through this pilot study, we would like to examine the issue of screening boys alone based on actual evidence, which will lead to a discussion on targets of sex for publicly funded screening. For example, a male patient diagnosed after the onset of the disease may not have benefited from early detection through family analysis of female carriers. Further discussion is also warranted regarding the justification for diagnosing PD with increased VLCFA in boys only. The reason for the latter may be that the disease is already present in the newborn period, or there is no effective treatment for these diseases; however, the diagnostic inequity by sex is a problem in this study. In the Netherlands, screening is suspended if ALD is ruled out in male patients with increased C26:0-LPC [22]. On the other hand, in the USA, further analysis continues for both sexes with increased C26:0-LPC, even after excluding ALD to avoid a diagnostic odyssey [18]. We would like to continue with the pilot study and further examine the issues identified.

## 6. Conclusions

Given that “pre-onset diagnosis leads to improved disease prognosis in male ALD patients”, it seems necessary to expand newborn screening to the rest of the country, incorporating a wide range of opinions from experts, patients, and bioethics groups. On the other hand, in response to the argument that it is difficult to predict the prognosis of male ALD patients even if the disease is detected by newborn screening, it should be necessary to develop a method to predict the disease type and prognosis. As newborn screening for ALD will spread throughout the world, it may be expected that many ALD patients will be detected before onset, resulting in improvement of their prognosis, and further elucidation of the pathogenesis will lead to the development of new treatments.

## Figures and Tables

**Table 1:** Various phenotypes of adrenoleukodystrophy.

Phenotypes	Onset	Symptoms	Japanese Patients (At Ddiagnosis by Gifu Univ.)
Childhood cerebral ALD (CCALD)	3–10 years	personality and behavioral changes, decreased vision and hearing, impaired intelligence, gait disturbance	99
Adolescent cerebral ALD (AdolCALD)	11–21 years	similar to CCALD but tends to progress more slowly.	12
Adult cerebral ALD (ACALD)	after adulthood	personality changes, intellectual deterioration, psychiatric symptoms	17
Adrenomyeloneuropathy (AMN)	after puberty	gait disturbance, incontinence, impotence, progresses slowly. Some patients may develop cerebral ALD.	55
Olivo-ponto-cerebellar type of ALD (OPCALD)	after adulthood	cerebellar ataxia, spasticity of the lower limbs, similar to spinocerebellar degeneration. Some patients may develop cerebral ALD.	6
Addison only	after infancy	lethargy, anorexia, weight loss, skin pigmentation Some patients may progress to cerebral ALD or AMN.	29
Presymptomatic		We cannot predict phenotypes and prognosis before onset.	43

## References

[1] Shimozawa N., Imanaka T., Shimozawa N. (2019). Peroxisomal disorders. Peroxisomes: Biogenesis, Functions, and Role in Human Disease.

[2] Shimozawa N., Tsukamoto T., Suzuki Y., Orii T., Shirayoshi Y., Mori T., Fujiki Y. (1992). A human gene responsible for Zellweger Syndrome that affects peroxisome assembly. Science.

[3] Peters C., Charnas L.R., Tan Y., Ziegler R.S., Shapiro E.G., DeFor T., Grewal S.S., Orchard P.J., Abel S.L., Goldman A.I. (2004). Cerebral X-Linked adrenoleukodystrophy: The international hematopoietic cell transplantation experience from 1982 to 1999. Blood.

[4] Takemoto Y., Suzuki Y., Horibe R., Shimozawa N., Wanders R.J., Kondo N. (2003). Gas chromatography/mass spectrometry analysis of very long chain fatty acids, docosahexaenoic acid, phytanic acid and plasmalogen for the screening of peroxisomal disorders. Brain Dev..

[5] Shimozawa N. (2011). Molecular and clinical findings and diagnostic flowchart of peroxisomal diseases. Brain Dev..

[6] Takashima S., Toyoshi K., Itoh T., Kajiwara N., Honda A., Ohba A., Takemoto S., Yoshida S., Shimozawa N. (2017). Detection of unusual very-long-chain fatty acid and ether lipid derivatives in the fibroblasts and plasma of patients with peroxisomal diseases using liquid chromatography-mass spectrometry. Mol. Genet. Metab..

[7] Takashima S., Saitsu H., Shimozawa N. (2019). Expanding the concept of peroxisomal diseases and efficient diagnostic system in japan. J. Hum. Genet..

[8] Shimozawa N., Imanaka T., Shimozawa N. (2019). Diagnosis of peroxisomal disorders. Peroxisomes: Biogenesis, Functions, and Role in Human Disease.

[9] Suzuki Y., Takemoto Y., Shimozawa N., Imanaka T., Kato S., Furuya H., Kaga M., Kato K., Hashimoto N., Onodera O. (2005). Natural history of X-linked adrenoleukodystrophy in Japan. Brain Dev..

[10] Engelen M., Kemp S., de Visser M., van Geel B.M., Wanders R.J., Aubourg P., Poll-The B.T. (2012). X-linked adrenoleukodystrophy (X-ALD): Clinical presentation and guidelines for diagnosis, follow-up and management. Orphanet J. Rare Dis..

[11] Regelmann M.O., Kamboj M.K., Miller B.S., Nakamoto J.M., Sarafoglou K., Shah S., Stanley T.L., Marino R. (2018). Pediatric endocrine society drug and therapeutics/rare diseases committee. Adrenoleukodystrophy: Guidance for adrenal surveillance in males identified by newborn screen. J. Clin. Endocrinol. Metab..

[12] Huffnagel I.C., Laheji F.K., Aziz-Bose R., Tritos N.A., Marino R., Linthorst G.E., Kemp S., Engelen M., Eichler F. (2019). The natural history of adrenal insufficiency in X-linked adrenoleukodystrophy: An international collaboration. J. Clin. Endocrinol. Metab..

[13] Mallack E.J., Turk B.R., Yan H., Price C., Demetres M., Moser A.B., Becker C., Hollandsworth K., Adang L., Vanderver A. (2021). MRI surveillance of boys with X-linked adrenoleukodystrophy identified by newborn screening: Meta-analysis and consensus guidelines. J. Inherit. Metab. Dis..

[14] Sawada T., Kido J., Yoshida S., Sugawara K., Momosaki K., Inoue T., Tajima G., Sawada H., Mastumoto S., Endo F. (2020). Newborn screening for Fabry disease in the western region of Japan. Mol. Genet. Metab. Rep..

[15] Sawada T., Kido J., Nakamura K. (2020). Newborn screening for Pompe Disease. Int. J. Neonatal Screen..

[16] Hubbard W.C., Moser A.B., Liu A.C., Jones R.O., Steinberg S.J., Lorey F., Panny S.R., Vogt R.F., Macaya D., Turgeon C.T. (2009). Newborn screening for X-linked adrenoleukodystrophy (X-ALD): Validation of a combined liquid chromatography-tandem mass spectrometric (LC-MS/MS) method. Mol. Genet. Metab..

[17] Hubbard W.C., Moser A.B., Tortorelli S., Liu A., Jones D., Moser H. (2006). Combined liquid chromatography-tandem mass spectrometry as an analytical method for high throughput screening for X-linked adrenoleukodystrophy and other peroxisomal disorders: Preliminary findings. Mol. Genet. Metab..

[18] Moser A.B., Jones R.O., Hubbard W.C., Tortorelli S., Orsini J.J., Caggana M., Vogel B.H., Raymond G.V. (2016). Newborn screening for X-linked adrenoleukodystrophy. Int. J. Neonatal Screen..

[19] Wiens K., Berry S.A., Choi H., Gaviglio A., Gupta A., Hietala A., Kenney-Jung D., Lund T., Miller W., Pierpont E.I. (2019). A report on state-wide implementation of newborn screening for X-linked adrenoleukodystrophy. Am. J. Med. Genet. A.

[20] Lee S., Clinard K., Young S.P., Rehder C.W., Fan Z., Calikoglu A.S., Bali D.S., Bailey D.B., Gehtland L.M., Millington D.S. (2020). Evaluation of X-linked adrenoleukodystrophy newborn screening in North Carolina. JAMA Netw. Open.

[21] Armangue T., Orsini J.J., Takanohashi A., Gavazzi F., Conant A., Ulrick N., Morrissey M.A., Nahhas N., Helman G., Gordish-Dressman H. (2017). Neonatal detection of Aicardi Goutieres Syndrome by increased C26:0 lysophosphatidylcholine and interferon signature on newborn screening blood spots. Mol. Genet. Metab..

[22] Barendsen R.W., Dijkstra I.M.E., Visser W.F., Alders M., Bliek J., Boelen A., Bouva M.J., van der Crabben S.N., Elsinghorst E., van Gorp A.G.M. (2020). Adrenoleukodystrophy newborn screening in the Netherlands (SCAN Study): The X-factor. Front. Cell Dev. Biol..

[23] Wilson J.M.G., Jungner Y.G. (1968). Principles and Practice of Screening for Disease.

